# Functional Diseases of the Eye

**Published:** 1872-10

**Authors:** Swan M. Burnett

**Affiliations:** Knoxville, Tenn.


THE GEORGIA
MEDICAL COMPANION:
A MONTHLY ADVISER.

VOL. 2. ATLANTA, GA., OCTOBER, 1872. NO. 10.

### PART I.

### Original Communications and Special Selections.

### TUNCTIONAL DISEASES OF THE EYE—MYOPIA.

BY SWAN M. BURNETT, M. D., KNOXVILLE, TENN.

The etymology of the word Myopia, makes it mean *mouse eyed* (*mus*, mouse and *ops*, eye), from a fancied resemblance of the eyes of myopes to those of the mouse. Donders, however, in his re-arrangement of the nomenclature of this department of Ophthalmology called this condition in accordance with its chief and constant characteristics and in keeping with the nomenclature of the other anomalies of refraction, *Brachymetropia*—(*brachus*, short, *metros* measure, *ops* eye), that is vision within the normal limits. The term myopia had been, however, so long in use that it has been thought unadvisable to change it for a new one, and it is still retained, as are many other terms, in medical literature, which have either no significance, or false ones. The prominent characteristic of M, is well known. Objects to be clearly distinguished must be brought close to the eye—(hence the term nearsightednsss), while those at a distance are seen but imperfectly. The farthest point of distinct vision lies within a few inches of the eye, instead of at an infinite distance, as we have seen it should do in the Emmetropic eye. We have also seen that in the normal eye parallel rays are focused upon the retina during rest of accommodation; in myopia, however, these rays, during rest of accommodation, are brought to a focus *before* the retina, and only those that are *divergent* are focused upon it.

The cause of this must evidently be one of two things: either there is too much refraction of the rays, or else the retina is removed to too great a distance from the refracting surfaces. Many and ingenious have been the theories advanced conserning

THE CAUSE OF M., based upon one or the other of the above principles. It has been attributed, variously, to an increased convexity of the lens, and in increase in the density, and consequently an increase of the index of refraction of the humors of the eye. In Dunglison's Medical Dictionary, in the edition so late as 1860, we find the following in the definition of Myopia: "The defect is owing to too great convexity of the eye, or too great density of the humors, and is palliated by wearing concave glasses." Displacements and a too forward position of the lens have also been assigned as causes.

Such were the opinions held until within a very recent period, and are even now commonly received by those who have not been keeping pace with the rapid advances of ophthalmology. The researches of Donders, however, made quite a revolution in our ideas respecting this, and as well as the other anomalies of refraction; and physiological and pathological optics generally.

Each of these opinions deserve at least a passing notice. By means of an apparatus of his own contrivance, called a *phacoido-scope*, Donders has been able to measure the radius of curvature of the cornea with great accuracy, and from this he is able to calculate the degree of convexity. From an examination of a large number of myopic eyes, he has established, beyond a doubt, that the convexity of the cornea is *not* increased, but on the contrary is *less* than in Emmetropia, and that the lessening of the corneal convexity is just in proportion to the degree of the myopia. From the fact that myopia is commonly met with in students and those who use their eyes a great deal for near work, it would be natural for us to infer that the strain upon the accommodation would have an effect to produce an increased convexity of the lens, and thus give rise to M. By means of the same apparatus Donders is also able to measure the curvature of its lens and he has never found it in any case of M—to be materially different from the normal. A too forward position of the lens has not been proven in M— but on the contrary, it is further removed from the cornea, even than in Hypermetropia, as is evidenced by the increased depth of the anterior chamber. As regards the increase in the density of the humors of the eye, the opposite condition has generally been found, at least in the higher degrees of M.

What then *is* the cause of Myopia? Here modern ophthalmology has cleared away the darkness and has shown positively that *Myopia depends upon an increase in the antero-posterior diameter of the eye.* This is proven by measurements of the eyes, both during life and after death.

We have said that in M., parallel rays are not brought to a focus upon the retina but before it, and that only *divergent* rays are properly focused. As to the

PATHOLOGY

of M—, our knowledge on some points is not positive. It is a question as to whether it is congenital and hereditary or acquired. Both views have strong and weighty authority for their support. In the great majority of instances, however, the facts and history of the case go to show that it is congenital, or at least that the predisposition to it, is. Von Ammon has pointed out some very interesting facts that make it highly probable that this predisposition is the result of a weakness at the port-pole of the eye, caused by an arrest of development. The elongation of the globe generally takes the form of a *saphyloma posticum*—that is, a bulging at the port portion of the eye. Von Grafe held to an inflammatory development of this staphyloma, and gave the name of *sclerotico-chorioriditis posterior* to the condition. It is true not many cases of M pass through their course without some symptoms of irritation or congestion, or even inflammation in the choroid, but then there are others which neither during life nor after death present any signs of them whatever.

Donders' conclusions are, "that almost without exception, the predisposition to the development of staphyloma posticum exists at birth; that it is developed with symptoms of irritation which, in moderate degrees of staphyloma do not attain any great clinical importance, but that in the higher degrees an inflammatory state almost always occurs, at least at a somewhat more advanced time of life, as a *result* and as a *co-operative cause of the further development* of the distension and of the atrophy.

Under this head we will speak of a peculiar and marked ophthalmoscopic appearance that is observed almost invariably in degrees of M. above 1-12. We allude to the characteristic crescent usually seen bordering the outer side of the optic nerve. It varies in size, generally in proportion to the degree of the myopia, from a mere line to a surface as large or even larger than the disc itself. It is readily distinguished from the nerve-surface by its dead white color, the retinal vessels run straight across it and show with great distinctness against the white back-ground. Paints of pigment are nearly always seen at the edges, and frequently on the surface. The form often varies, sometimes extending entirely around the nerve becoming *annular*. White atrophic patches are also seen frequently in other parts of the fundus. These appearances are the result of an atrophy of the choroid, and they furnish corroborative evidence of the existence of staphyloma posticum. As a consequence of the elongation of the globe the tissues are put very much upon the stretch, and the choroid being the most inelastic, gives way, becomes atrophied and exposes the white sclerotic behind. Its attachment to the optic nerve-entrance being very slight, it, of course, gives way there first, and we have the white crescent above alluded to. It will be seen that the size of the crescent is in some degree a measure of the extent of the staphyloma. If the staphyloma is very great, the retina gives way too and becomes detached, and we then have one of the most serious and pernicious complications of M. We will speak of this further on when considering the results of M.

It is very important that the

DEGREE OF MYOPIA

be correctly ascertained. There are two methods for accomplishing this: 1st, by means of suitable test glasses, and by the ophthalmoscope. The latter, however, is only successful in the hands of experts, and we will not here enter into a full description of the manner in which it is done; suffice it to say that it is calculated from the distance at which a reverse image is clearly seen in the direct method, and also by the strength of the concave glass that it is necessary to use behind the mirror to obtain a well-defined, upright image of the fundus at the distance ordinarily used for making observations in this method.

It has this great advantage, that we are able to use it in the case of young children and those on whose statements we cannot rely.

The simpler method and the one adapted to the majority of cases is the use of the test glasses (concave) and Snellen's test-types. The test-types of Snellen consist of a combination of testers arranged in rows upon a board or sheet, whose size increases from the bottom to the top, each row being a little larger than the one below. There are generally seven rows, and they are numbered according to their size from CC (200) at the top, to XX (20) at the bottom. They are thus numbered from the fact that normal eyes should be able to read them at the distance in feet of each number, without the intervention of any glasses. Thus they should read C C, at 200 feet; X X, at 20 feet, and so on. They are intended to be of such a size that those distances rays of light emenating from them will strike the cornea under an angle of 5 degrees which is supposed to be the limitry angle of distinct vision. The test glasses should, of course, be concave, and are to be found in the cases of "test glasses," obtained from the opticians. As these cases, when complete, are rather expensive, none but specialists generally have them. At the last meeting, however, of the German Ophthalmological Congress, at Heidelberg, a set of glasses was introduced, consisting of but 8 concave and 8 curve glasses, which, by various combinations can be made applicable to all ordinary cases. These will certainly be of great convenience to the general practitioner.

In order to find the degree of M., place the patient at 20 feet distant from the test-types and see how far down he can read. You will thus become aware of how imperfect his vision is for distance. Often you will find him scarcely able to distinguish C C, at 20 feet. Then see the greatest distance at which he can read the smallest type (brilliant) and mark that. This will give you a clue to his degree of M., for it marks pretty accurately his farthest point of distinct vision and will suggest the proper glasses, approximately at least. Say, then, that he can read it at 6 inches. You apply a concave lens of 6 inches (negative) focal length, and let him read down the test-types as far as he is able; then try a 7 inch, and then a 5 inch and see with which he is able to see with the greatest distinctness; this when found, will mark the degree of his M. It is sometimes a little difficult to decide between two glasses. He is unable, say, to decide between a 6 and 7. Having the 6 before his eyes, you apply before that a weak concave glass, say, 70 and if vision is not improved the 6 is too strong, and if, on applying a weak convex lens of the same strength vision is improved, we may know that the proper glass is between a 6 and 7.

Now, what have we discovered when we have found the degree of M, and what do we mean when we say a patient has M—1-6?

We have discovered that 6 inches is the farthest point at which he is able to see objects plainly during rest of accommodation, and that rays of light in order to be properly focused upon the retina must assume a direction as though they diverged from a point situated 6 inches in front of the eye.

On examination, we generally find myopic eyes to be rather prominent, and the anterior chamber will nearly always be found deeper than in the normal eye. If there is any considerable staphyloma posticum we readily see the evidences of it when the eye is turned strongly inwards, the depression or hollow usually found between the outer canthus and the globe having disappeared. Myopes, too, often bring the eye-lids close together, which gives them a characteristic appearance. This is done in order to cut off as many of the rays of dispersion as possible and thus make the image more clearly defined.

The influence of M. upon the general character of the individual should not be overlooked. One can scarcely have failed to notice the vacant look about the face of a myope; they very seldom fix the person whom they address, which gives them an inattentive, careless appearance. From the fact that they have no correct idea of how they appear to others, their manner shows a freeness and degree of self-confidence; or, on the other hand, a bashfulness and backwardness that is by no means a true exponent of their character. To the myope, whose defect is uncorrected, a large amount of the beautiful in nature and art is lost. His world is, as it were, circumscribed, its extreme limit not extending beyond a few inches, or at most a few feet. From this cause it has been said that myopes are particularly amorous, for not being able to observe the faults and defects of human beings, they look upon them as angels. Many are not conscious of the degree of their imperfection of vision and pass their whole lives in a darkness that might otherwise be made a glorious light to them. Physicians, as a rule, are not considerate enough about this. The myope, not being thoroughly conscious of the degree and amount of his affliction, make little or no complaint, and his physician, because perhaps, its investigation would entail some labor and research, passes by his case with an indifference and carelessness that is little short of culpable. The

RESULTS AND COMPLICATIONS

of M. deserve careful consideration, as they are often very fatal to vision, and because they can, if the proper measures are instituted sufficiently early, generally be prevented.

Prominent among them is *strabismus.* Donders has established the rule, that while we find *strabismus convergens* associated with hyperopia, *strabismus divergens* nearly always has myopia as a cause. Its origin may be either mechanical or functional. The mechanical form is the result of the staphyloma posticum which prevents the free rotation of the globe in the socket, and consequently a proper convergence of the optic axies. If it is extensive the globe becomes almost fixed. In this case the globe taking the direction of the axis of the orbit is slightly converged and we thus have the apparently inconsistent anomoly of strabismus divergens for near, and strabismus convergens for distant vision.

This condition is, of course, only found in very high degrees, but we often find a strabismus in the lower degrees, which is of a *functional* origin. Even in these lower degrees of M. the patient has to approximate the object more closely than normal, and this, of course, entails a greater degree of convergence.

A great strain is thus put upon the int. recti muscles. If these muscles happen to be weak, one of them is very apt to give way under the strain, and the eye is allowed to deviate, possibly outwards; the patient being willing to sacrifice binocular vision for freedom from pain and for the privilege of a nearer approximation of the object, and consequently a greater distinctness of the image. Any cause that makes live ocular vision imperfect, as anopacity of one cornea, or a difference in the refraction of the two eyes will produce the same result. There is a natural tendency, however, to preserve tenocular vision and the effort to do this often gives rise to a very considerable amount of pain, which is called *asthenopia.* This form of painful vision is called *muscular* in contradistinction to the asthenopia of hyperopia, though why I am unable to see since both undoubtedly depend upon muscular fatigue; the latter of the ciliary muscle, and the former of the internal recti. In the asthenopia of M., the pain is referred to the eye and generally to the muscles affected, whereas in H. it is referred to the brow, forehead and adjacent parts. It would be a very natural supposition that in strabismus we would have double vision. Such, however, is seldom the case, from the fact that the image of the deviating eye is *suppressed.* An image, it is true, is formed upon the retina, but it is utterly ignored by the nervous centre of vision. It is a purely psychical act, and if continued for any length of time the eye loses very considerably the power of perceiving images. With practice, however, this will soon return.

Another serious complication of M. is *detachment of the retina.* It is found most generally in the high degrees, and the manner of its production will be readily seen, when we consider the excessive stretching it, as well as the other tunics of the eye must undergo in an extensive staphyloma posticum. Being firmly united to the nerve it can not give way there or does the choroid, neither can it become detached at the ora serrota—so when it does yield to the strain it must become detached about the equator. We then see it as a thin film floating about in the vitreous, generally funnel-shaped with the apex at the nerve. It is distinguished from detachment of the hyaloid by the vessels which are still visible on it If the detachment take place in the vicinity of the yellow spot centre vision is no longer possible. The visual field is always limited or interrupted, and such limitations or interruptions generally mark pretty accurately the extent and character of the detachment. This condition, which is irremediable, is most deplorable indeed, and the prospect for the future any thing but cheering, as there is always a tendency to an increase of the detachment.

Myopic eyes, especially after use for close work for any considerable length of time, show signs of *irritation* or even the more active powers of inflammation. They become sensitive to light—there is injection of the conjunctiva and sclera, and vision is very much impaired. If we examine the fundus with the opthalmoscope at this time, we will find a very considerable hyperæmia, the vessels of the disc will be found to be larger and increased in number, and the edges of the crescent will show marked evidences of congestion. This is the condition described by Von Grafe, as *sclerotico chorioriditis posterior*, and under it the M. shows a marked tendency to increase, for even after all signs of congestion and inflammation have subsided, the vision is never so good as before, and an examination will generally show that the M. has progressed.

### PROGNOSIS.

Donders, by observing a large number of cases, for a long time, and making accurate examinations at intervals, has established the law of a steady increase of M., though it may be so slight as to be scarcely perceptible. Myopia, however, is for practical purposes, divided into *stationary*, *temporarily progres-*

*sive*, and *permanently progressive.* Even in the stationary form there is a disposition to an increase about the period of puberty, and it is at this time that the increase is most marked in the temporarily progressive form. By suitable treatment and proper hygienic means it may be arrested here and remain stationary from this time forward. In the majority of cases, however, it is permanently progressive, though its progress, after this time, is not so rapid or marked as before. And here it will be well enough perhaps, to speak of an error not confined altogether to the laity, viz: that a myopic eye gets stronger and more useful with age, and that the myope at an advanced time of life will be happily able to dispense with glasses, such as common presbyopes use. Such a belief is fraught with much evil, for it lulls the myope into an indifference to his trouble, holds out the delusive hope of improvement with time, and thereby prevents his taking the proper means for its correction. With very low forms of stationary M. it is true that as the accommodative power begins to give way with age, there will be a compensation in the error of refraction, and the waring of glasses may thus be postponed a number of years perhaps. Such cases are, however, rare, and we can never, in practice, be certain of them, for we must remember that the myopic eye is a *diseased* one, and that we are never able to say that the condition will not increase. If, however, the myopia is, early in life, neutralized by proper glasses, and proper hygienic means are instituted, we can fairly promise that there will be but a slow, if any increase of the M. Many of the distressing symptoms will be avoided, and if the myopia is not of high degree vision will not be materially different from that of Emmetropes. The glasses applied become a part and parcel of the refracting apparatus of the eye, the accommodation adapts itself to them, and if the M. does not increase, the same glasses may be used from youth to the time when age begins to affect the accommodation. Most all the serious complications of the malady are also avoided by these means, and the great importance of an early institution of them is thus manifest. We now approach a most important part of the subject, and one of the most practical importance. "In the

TREATMENT

of myopia," says Donders, "the task of the oculist resolves itself into the following: 1st. To prevent the further development of the myopia and the occurrence of secondary disturbances. 2d. By the use of suitable glasses to render the use of myopic eyes easier and safer. 3d. To remove the asthenopia muscularis by the use of glasses or by tenotomy. 4th. To combat secondary disturbances of M."

To fulfill the first indication, two things must especially be avoided: *strong convergence of the optic axies and a stooping posture.* Many myopes labor under the delusion which is encouraged by many in the profession, that the use of glasses *is detremental* to the sight, and that the eyes will be strengthened by use without them; consequently they put off the procuring of suitable glasses as long as possible and continue to approximate objects close to the eye, and in reading and writing to bend over their work—two things they ought by all means not to do. These are two of the most active factors in the production of an increase of M., and in the following manner: During strong convergence there in an increased tension of the ocular muscles especially of the internal recti. A strong pressure is thus exerted upon the globe, and such is the origin and insertion of the muscles that the greatest amount falls upon the equator and tends to produce a flattening at that point and a consequent elongation of the antero-posterio diameter of the globe. The already weakened condition of the posterio pole of the eye makes it the more susceptible to this influence and an increase of the posterior staphyloma is the almost inevitable consequence. The stooping posture tends to keep up a congested condition of the fundus by preventing a free return of blood from the eye. This produces a softening of the tissues already too weak, or still further increases the liability to an aggravation of the staphyloma. The eyes, for near work, should be fitted with spectacles of such strength as to bring the farthest point to the ordinary reading distance, say 16 to 18 inches. Strong convergence is thus avoided and the patient is relieved of the necessity of stooping and bending over his work. When there is a deviation of one eye, there is, of course, no pressing necessary for this, as in that case there is no convergence, the patient fixing with one eye only.

Myopes should always read with the head well held up, and should use a high desk for writing, and should, by all means avoid reading in a reclining position.

It is highly important that the glasses should be properly adapted, else the trouble may be aggravated instead of being relieved. The myope should always consult a competent medical man and have the exact state of his refraction ascertained. The proper glassess for its correction will then be prescribed, just the same as a remedy will be prescribed for a derangement of any other bodily function. If he goes to a jeweler or even an optician and selects a pair at hap-hazard, he is apt to do his eyes more damage than good. We should beware of giving too *strong* glasses in M. as we would in giving too *weak* ones in H. Too strong glasses, bring the accommodation into play and occasion a straining of the eyes that is highly detrimental. So it is better to err on the opposite side and leave the other, the myopia, not quite neutralized. But of course glasses are mostly given to procure distinct vision of distant objects, near ones being seen with sufficient accuracy, and it is only in high degrees and in exceptional cases that we have to use them for near objects too. The question may be asked how do concave glasses correct myopia, and give a distinct image of distant objects? In the myopic eye we see that the parallel rays are, by the simple refraction of the eye brought to a focus before the retina, because the retina is removed further back from the refracting surfaces than in the Emmetropic eye; but if those rays can be made to diverge as though they came from a point situated just in front of the eye at the greatest distance of distinct vision, they will be brought to a focus upon the retina and there will be a well-defined image. This can be done by means of a concave lens placed in front of the eye, and the lens must be of just that strength that will make these parallel rays diverge after passing through it as though they came from the furthest point of distinct vision in that particular case—if the furthest point was 8 inches, the lens must cause the rays to diverge as if they came from 8 inches before the eye, if it is 6 inches, the lens must, if 6 inches negative, focal length, and so on. The M. is thus completely neutralized and the eye is (theoretically) Emmetropic, for the far point now lies at an infinite distance, and parallel rays are brought to a focus upon the retina during rest of accommodation. This is actually the case if the glasses are applied sufficiently early in life, before the relation between convergence and accommodation has been disturbed. But if the myope puts off the wearing of glasses until he is somewhat advanced in life, this is not the case, for the accommodation has undergone such a modification by that time that it will not allow the same glasses to be used for near and distant vision. Consequently he must be provided with two pair, often, one completely neutralizing his M., for distant vision, and one that will bring the far point to the distance at which he may want to use his eyes—if for reading at 16 to 18 inches, if for lecturing, preaching, etc., at about 20 to 24 inches, and so on. The advantage of an early adaption of glasses are thus evident. By so doing we place myopic children more nearly upon a level with Emmetropes and by proper hygienic management can almost certainly prevent many of the disastrous results and complications spoken of in a previous section. The *asthenopia muscularis* is often completely relieved, when by wearing suitable glasses the far point is removed farther away and the strain on the internal recti thus taken off.

But we often meet with cases where the strabismus has already become fixed, or where the asthenopic has failed to be relieved by glasses and properly instituted hygienic measures. Here the propriety of division of the external recti comes in for consideration. In a great many cases, a division of the external rectus restores the equilibrium and balance of muscular power and the troublesome symptoms are at once and permanently relieved. We are not so fortunate, however, in all cases, nor is it even desirable, under all circumstances, to resort to the operation. The effect of prismatic glasses should first be tried.

Grafe advocated a combination of prismatic and concave glasses, as an excellent means of restoring binocular vision and relieving asthenopia. If these fail, he has laid down rules with great precision for the performance of tenotomy, which are, however, too lengthy to re-produce here.

The management of the eyes during the attacks of irritation and inflammation is of the greatest importance. These attacks, as we have before remarked, are most frequent about the period of puberty, and it is at this time that the eyes of myopes should be watched with the most sedulous care. So soon as the eyes begin to show the least signs of irritation *all* work must be suspended *at once*, cold douches must be applied to the eyes and all bright lights avoided. The feet must be kept warm. These means will often suffice to dissipate the trouble and the patient may be allowed gradually to resume near work—always taking care, however, to give the eyes a rest every 15 or 20 minutes.

If the symptoms are more marked, more active means must be instituted—the artificial leech must be applied to the temples, the patient confined to a dark room, salines given, and the diet restricted. For those melancholy complications, detachment of the retina and suffusion of blood, unfortunately we are almost powerless to afford a remedy. The blood will, to some extent, be absorbed, but it leaves membranous opacities behind that always seriously impair vision. Puncture of the retina has been recommended in detachment, but the results of the operation are anything else but satisfactory. In strabismus where it is not deemed advisable to operate, it is highly important that the power of vision in the denoting eye, should be retained, and for this purpose it is advisable to practice it for a while every day, the other being closed.

